# Thermometer at Nashville

**Published:** 1870-05

**Authors:** W. H. Morgan


MR. EDITOR:—I forward you this table for publication in the REGISTER. It is sent you at the request of several members of the American Dental Association who are afraid of the heat in this locality in August.

Very Truly, W. H. MORGAN.

| | | 6 A.M. | 2 P.M. |
|---|---|---|---|
| | August 1 - - - | 69 | 84 |
| | " 2 - - - | 70 | 82 |
| | " 3 - - - | 68 | 86 |
| THERMOMETER AT NASHVILLE. | " 4 - - - | 76 | 89 |
| | " 5 - - - | 78 | 90 |
| | " 6 - - - | 62 | 79 |
| *From Aug. 1st to 14th,* 1869. | " 7 - - - | 60 | 77 |
| | " 8 - - - | 59½ | 80 |
| | " 9 - - - | 60 | 82 |
| | " 10 - - - | 62 | 84 |
| | " 11 - - - | 68 | 86 |
| | " 12 - - - | 76 | 88 |
| | " 13 - - - | 75 | 89 |

